# Pedagogical GenAI use and self-reported creative cognition in art education: a three-wave time-lagged correlational study among design students

**DOI:** 10.3389/fpsyg.2026.1916135

**Published:** 2026-10-06

**Authors:** Jingyi Zhang, Da Xing

**Affiliations:** 1 Department of Fashion Design, Pukyong National University, Busan, Republic of Korea; 2 School of Information Technology and Engineering, Guangzhou College of Commerce, Guangzhou, China

**Keywords:** AI-related learning anxiety, art education, creative engagement, creative self-efficacy, design students, generative artificial intelligence, self-reported creative cognition, time-lagged correlational study

## Abstract

Generative artificial intelligence (GenAI) is increasingly used in design education, yet the psychological pathways associated with students' self-reported creative cognition remain unclear. In this three-wave time-lagged correlational study, 389 design students at a South Korean university reported pedagogical GenAI use at Wave 1, creative self-efficacy and AI-related learning anxiety at Wave 2, and creative engagement and self-reported creative cognition at Wave 3. Pedagogical GenAI use referred to coursework-related use for ideation, style exploration, revision, comparison of alternatives, and feedback seeking. Covariance-based structural equation modeling showed good fit [comparative fit index (CFI) = 0.983, root mean square error of approximation (RMSEA) = 0.026]. Higher GenAI use was associated with higher self-efficacy (β = 0.489) and lower anxiety (β = −0.191). The total modeled association with self-reported creative cognition was β = 0.456 [95% CI [0.314, 0.604]]; its indirect components constituted approximately 59%. The GenAI-origin serial indirect association through self-efficacy and engagement was β = 0.122, whereas the corresponding anxiety-engagement association was β = 0.015. Separately, self-efficacy was indirectly associated with creative cognition through engagement (β = 0.249); this quantity does not include the preceding GenAI-use path. Because the study was observational and engagement and cognition were measured concurrently at Wave 3, the findings do not identify causal mediation. They are consistent with an appraisal-to-engagement account in which competence beliefs have a larger modeled role than anxiety reduction.

## 1 Introduction

Generative artificial intelligence (GenAI) now sits inside the design studio. Text-to-image systems, prompt-based editors, and conversational assistants supply visual references, style variations, and written critique on demand, and design and art programs have folded them into briefs, crits, and portfolio work. This extends a longer line of work on technology and creativity in education (Henriksen et al., 2016) into a setting where the tool generates candidate content rather than only supporting the student who makes it, a shift that has reframed debates about authorship and learning (Dwivedi et al., 2023; Vinchon et al., 2023). Controlled evidence shows the stakes are not neutral. When writers could draw on a GenAI-generated opening idea, evaluators rated their stories as more creative and better written, with the largest gains among the least creative writers, even as the resulting stories grew more similar to one another (Doshi and Hauser, 2024). The same tension reaches ideation: large language models lift the fluency of individual idea generation while compressing the diversity of ideas across people (Anderson et al., 2024). For art education the practical question is therefore not whether students use these tools, but what happens to their creative thinking when they do. Design programs have a direct stake in the answer, because creative cognition is the competence their degrees certify and the one most plausibly reshaped by a system that returns finished-looking work in seconds.

Recent studies in design, art, and broader educational settings indicate that GenAI can support motivation, self-efficacy, feedback, and creative products, but that these outcomes depend on instructional structure and that perceived gains may diverge from objectively assessed creativity (Chen et al., 2025; Lu et al., 2025; Schmitt-Fumian et al., 2025; Cui and Li, 2026). Therefore, pedagogical GenAI use is defined as students' self-reported, coursework-related use of generative tools for visual ideation, style exploration, prompt-based revision, comparison of alternatives, and feedback seeking, rather than as general AI attitudes, technical proficiency, or logged platform behavior. The most directly comparable study surveyed 121 design students on a single occasion and reported a strong association between GenAI use and creative cognition (β = 0.61), partly accompanied by self-efficacy and lower anxiety (Hwang and Wu, 2025). A parallel cross-sectional result among engineering students linked GenAI use to creativity through AI self-efficacy and critical thinking (Zhou et al., 2025). Such single-occasion designs measure predictor, mediator, and outcome together, which prevents any separation of their temporal order and can inflate associations through shared method variance (Podsakoff et al., 2003).

These constructs are organized as a social-cognitive, appraisal-to-engagement account rather than a sequence of interchangeable mediators. GenAI-supported alternatives and feedback may be associated with creative self-efficacy, a belief about one's own capability, whereas concerns about dependence, originality, or keeping pace may be associated with AI-related learning anxiety, an affective appraisal narrower than global apprehension toward AI (Wang and Wang, 2022). These two appraisals are functionally distinct and need not lie at opposite ends of a single dimension. Both appraisals are expected to be associated with creative engagement, the behavioral, emotional, and cognitive investment a student brings to creative tasks (Fredricks et al., 2004; Zhang and Bartol, 2010), which in turn is associated with self-reported creative cognition, defined here as reported generative, exploratory, and combinatory activity rather than performance on an objective creativity task (Finke et al., 1992; Runco and Jaeger, 2012; Amabile, 1996). Self-efficacy is a motivational belief, not a cognitive act (Bandura, 1997; Beghetto, 2006); engagement is theorized as the behavioral enactment of the appraisal. Relative to Hwang and Wu (2025), the proposition tested here is thus not the addition of another mediator but an appraisal-to-engagement account in which competence and anxiety act as parallel appraisals with potentially unequal downstream roles and engagement represents their behavioral enactment.

Figure 1 summarizes this hypothesized three-wave associational model. Accordingly, we hypothesize that GenAI use is associated with higher self-reported creative cognition (H1). Creative self-efficacy accounts for part of that association (H2), and AI-related learning anxiety accounts for part of it such that lower anxiety accompanies higher cognition (H3). Creative engagement conveys the association from self-efficacy to cognition (H4). Finally, GenAI use is associated with cognition through a serial chain running from the two Wave-2 appraisals, through engagement, to cognition, separately for a competence branch (H5a) and an anxiety branch (H5b).

**Figure 1:**
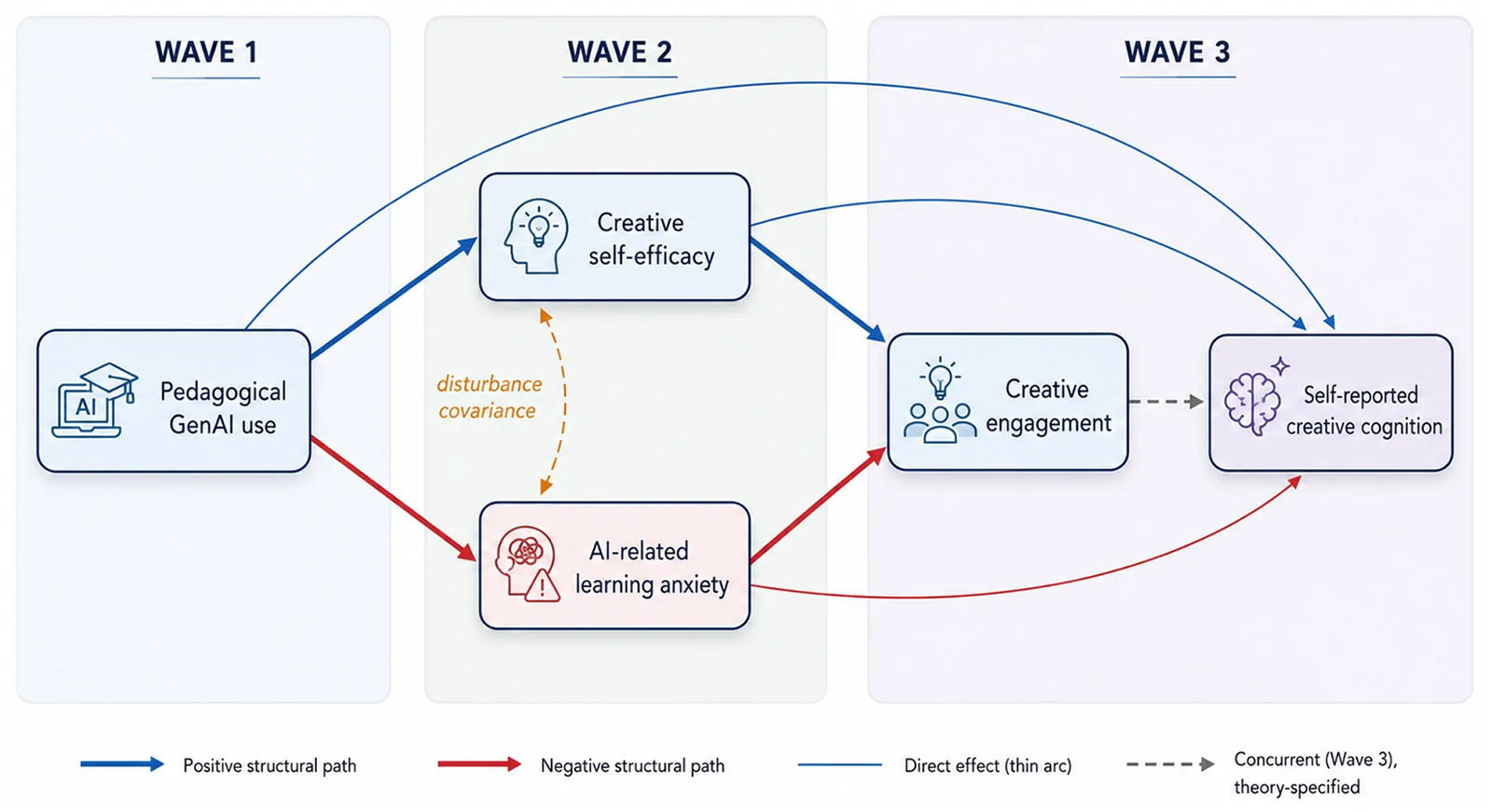
Hypothesized three-wave associational model (*N* = 389). Pedagogical GenAI use is measured at Wave 1; creative self-efficacy and AI-related learning anxiety at Wave 2; creative engagement and self-reported creative cognition at Wave 3. Colored solid arrows denote the theory-specified structural associations (blue positive, red negative) and the curved double-headed arrow the covariance between the two Wave-2 appraisals; thin arcs are the direct effects estimated with the mediators included, and the dashed arrow marks the engagement-to-cognition link, which was measured concurrently at Wave 3 and whose direction is theory-specified rather than temporally identified. Standardized coefficients are reported in Tables 3, 4, and the complete measurement model with standardized loadings appears in Supplementary Figure S1.

These hypotheses are examined with a three-wave, time-lagged correlational design that separates the predictor at Wave 1, the two intervening appraisals at Wave 2, and engagement and self-reported creative cognition at Wave 3. This places GenAI use before the mediators and the mediators before the outcome, which reduces same-occasion overlap for those links and orders them in line with the hypothesized process, but does not manipulate GenAI use or adjust for baseline levels of the mediators and outcome, so all paths are interpreted as associations rather than causal effects. Creative engagement and self-reported creative cognition were assessed together at Wave 3, so the engagement-to-cognition association is concurrent rather than time-lagged; its ordering is theory-guided rather than temporally identified, and reverse and non-directional specifications of this final link are examined. The sample comprises design students whose coursework routinely involves GenAI tools. Covariance-based structural equation modeling estimates the measurement and structural models jointly, and Monte Carlo confidence intervals evaluate the indirect associations.

## 2 Materials and methods

### 2.1 Participants and procedure

Participants were undergraduate students enrolled in design-related majors at a public university in Busan, South Korea, where generative tools are part of studio and digital-media coursework. Data were collected in three waves spaced approximately 2 weeks apart during one semester. At Wave 1, 452 students reported their pedagogical GenAI use and background characteristics. At Wave 2, 2 weeks later, they reported creative self-efficacy and AI-related learning anxiety. At Wave 3, they reported creative engagement and self-reported creative cognition. Responses across waves were linked through self-generated anonymous codes. Of the 452 Wave-1 respondents, 431 completed Wave 2 and 410 completed Wave 3, and 399 records matched across all three waves. From these matched records, further 6 cases were excluded that failed an embedded attention-check item and 4 cases flagged for careless responding by a combined long-string and response-time screen, leaving 389 cases with complete item-level data for analysis, a retention of 86.1% of the Wave-1 sample. The exact screening items, careless-response criteria, recruitment window, course context, and incentive information are reported in Supplementary Table S1, and the full participant flow appears in Supplementary Figure S2. The 2-week spacing between waves was chosen to be long enough to weaken same-occasion response tendencies yet short enough that the semester's coursework context stayed stable. Retained and attrited participants did not differ meaningfully on Wave-1 GenAI use, gender, year of study, or prior tool experience (all standardized mean differences < 0.15), and a logistic attrition model relating retention to these Wave-1 variables was non-significant (Nagelkerke *R*^2^≈0.01; Supplementary Table S1), so the matched sample is unlikely to be selective on the study variables.

Of the 389 participants, 251 (64.5%) were women and 138 (35.5%) were men, with a mean age of 21.4 years (*SD* = 1.9, range 18–27). Majors spanned fashion design (28.5%), visual communication design (26.0%), digital media and animation (24.4%), and industrial and product design (21.1%). Most reported more than 1 year of prior experience with generative tools (44.2%), with the remainder split between 6 and 12 months (33.4%) and under 6 months (22.4%).

### 2.2 Measures

All constructs were measured with multi-item seven-point Likert scales (1 = strongly disagree, 7 = strongly agree). The measures differed in origin: creative self-efficacy was adapted from the scale of Tierney and Farmer (2002), AI-related learning anxiety from the learning-anxiety subscale of the AI Anxiety Scale (Wang and Wang, 2022), and creative engagement was assembled from engagement and creative-process-engagement instruments (Fredricks et al., 2004; Zhang and Bartol, 2010), whereas pedagogical GenAI use and self-reported creative cognition were study-specific measures developed for this coursework context and informed by, rather than adapted from, existing theory. The outcome is termed self-reported creative cognition because its items assess reported generative, exploratory, and combinatory activity rather than performance on an objective creativity task, and a student's report that they generate or combine ideas may partly reflect perceived competence. The questionnaire was prepared in Korean through translation and back-translation. Complete Korean and English items, the source-to-item adaptation for each scale, translator qualifications and independence, the expert-review procedure, pilot statistics from 38 students, and a *post-hoc* content-classification audit by independent experts are provided in Supplementary Tables S2, S3.

*Pedagogical GenAI use* was measured with five items capturing how students use generative tools in coursework, including visual ideation, style exploration, prompt-based revision, and seeking feedback on generated alternatives. A sample item is “I use generative AI tools to explore alternative visual styles for my design assignments.” The measure assessed the extent of coursework-related functional use rather than general AI attitudes, technical proficiency, or logged behavior, so a one-point increase represents a one-point increase in the mean of five seven-point items covering these functions. The survey recorded functions of use but did not record platform-specific tools; tool-level frequencies are therefore unavailable, and the single score aggregates heterogeneous functions and should not be read as a platform-invariant treatment.

*Creative self-efficacy* was measured with four items adapted from Tierney and Farmer (2002), capturing confidence in generating, developing, and refining creative ideas in design tasks.

*AI-related learning anxiety* was measured with four items adapted from the learning-anxiety subscale of the AI Anxiety Scale (Wang and Wang, 2022), reworded for creative coursework. The items address worry about over-reliance on tools, about the originality of assisted work, and about keeping pace with tool use, and exclude job-replacement and sociotechnical content.

*Creative engagement* was measured with six items spanning behavioral, emotional, and cognitive investment in creative tasks, including absorption in the work akin to flow (Csikszentmihalyi, 1990), adapted from work on engagement (Fredricks et al., 2004) and creative process engagement (Zhang and Bartol, 2010).

*Self-reported creative cognition* was measured with five items reflecting reported idea generation, conceptual combination, and divergent exploration in design work, developed for this study and informed by the creative-cognition framework (Finke et al., 1992) rather than adapted from a validated psychometric scale.

### 2.3 Analytic strategy

Analyses used covariance-based structural equation modeling in R with the lavaan package (Rosseel, 2012) and robust maximum likelihood (MLR) estimation, with the seven-point items treated as continuous and each latent variable scaled by fixing its first loading. A two-step approach was followed: a confirmatory factor analysis established the measurement model, after which the structural paths were estimated. The analytic sample of 389 had no item-level missingness, so complete-case estimation was used, and the Wave-2 disturbances of creative self-efficacy and learning anxiety were allowed to covary. Model fit was judged against established benchmarks, with the comparative fit index (CFI) and Tucker–Lewis index (TLI) at or above 0.95, the root mean square error of approximation (RMSEA) at or below 0.06, and the standardized root mean square residual (SRMR) at or below 0.08 (Hu and Bentler, 1999); robust (scaled) test statistics and fit indices are reported, and standardized coefficients use the std.all solution. Convergent validity was assessed through composite reliability, McDonald's omega, and average variance extracted (AVE), and discriminant validity through the Fornell-Larcker and heterotrait-monotrait criteria (Fornell and Larcker, 1981). Indirect associations were tested with Monte Carlo confidence intervals based on 200,000 draws computed from the full estimated covariance matrix of the path coefficients, which perform comparably to bias-corrected bootstrapping and are well-suited to latent-variable mediation (Preacher and Selig, 2012; Preacher and Hayes, 2008; Hayes, 2018). Common-source variance was examined with a common-latent-factor model in which every indicator loaded on both its trait and an orthogonal method factor (Supplementary Table S5), and Harman's single-factor test retained only as an ancillary diagnostic (Podsakoff et al., 2003). Age, gender, year of study, major, and prior duration of GenAI use were included on theoretical grounds in a fully adjusted model (Supplementary Table S9) rather than selected by their effect on the structural coefficients. A model-based Monte Carlo analysis (10,000 replications) using the fitted paths, reliabilities, and estimator evaluated statistical power and interval coverage for each principal indirect association (Supplementary Table S8). Alternative specifications-a direct GenAI-to-engagement path, reverse and non-directional Wave-3 models, WLSMV re-estimation, non-parametric bootstrap intervals, and exclusion of the lowest-loading cognition item-are reported in Supplementary Tables S4, S7–S10, and a partial-least-squares path model on composite scores is included only as a supplementary estimator comparison (Supplementary Table S10).

## 3 Results

### 3.1 Measurement model

The five-factor measurement model fit the data well, χ^2^(242) = 306.40, *p* = 0.004, χ^2^/*df* = 1.27, CFI = 0.983, TLI = 0.981, RMSEA = 0.026 [90% CI [0.016, 0.035]], SRMR = 0.044. A one-factor alternative fit poorly by comparison, χ^2^(252) = 1961.1, CFI = 0.567, RMSEA = 0.132, which supports the five-factor structure and serves as a further check against common-method bias. Standardized loadings ranged from 0.60 to 0.80 and were significant; item-level loadings, item means, distributional statistics, composite reliability (0.81–0.88), McDonald's omega (0.81–0.88), and Cronbach's α (0.81–0.88) are reported in Table 1. AVE reached or exceeded 0.50 for four constructs; for self-reported creative cognition it was 0.496, marginally below the threshold, and together with that scale's lowest standardized loading of 0.602 this indicates marginal rather than unequivocal convergent validity, although composite reliability (0.83), omega (0.83), and a square-root AVE above all of its inter-construct correlations remained acceptable (Fornell and Larcker, 1981). The square root of each AVE exceeded the construct's correlations with the others, and every heterotrait-monotrait ratio stayed at or below 0.69, well under the 0.85 criterion (Table 2); discriminant validity therefore held under both criteria. A common-latent-factor sensitivity attributed about 8% of indicator variance to the method factor and shifted the focal structural coefficients by at most 0.03 (Supplementary Table S5); this reduces but does not eliminate concern about common-source variance, particularly because the outcome is self-reported and engagement and cognition were measured concurrently, and Harman's single-factor result (30.5% of variance) is reported only as an ancillary diagnostic.

**Table 1:** Measurement model: item statistics, standardized loadings, and construct reliability (*N* = 389).

Item	*M*	*SD*	Skewness	Kurtosis	λ
**Pedagogical GenAI use (**α = 0.839, ω = 0.840**, CR** = 0.839**, AVE** = 0.511**)**					
GAIU1	4.85	1.25	−0.24	−0.08	0.792
GAIU2	4.86	1.24	−0.21	−0.04	0.697
GAIU3	4.84	1.24	−0.21	−0.25	0.667
GAIU4	4.87	1.24	−0.22	−0.26	0.688
GAIU5	4.81	1.24	−0.06	−0.39	0.725
**Creative self-efficacy (**α = 0.858, ω = 0.859**, CR** = 0.858**, AVE** = 0.602**)**					
CSE1	4.60	1.25	−0.03	−0.29	0.801
CSE2	4.63	1.24	−0.14	−0.34	0.785
CSE3	4.64	1.26	−0.06	−0.42	0.764
CSE4	4.62	1.28	−0.06	−0.54	0.754
**AI-related learning anxiety (**α = 0.810, ω = 0.811**, CR** = 0.810**, AVE** = 0.516**)**					
AILA1	3.42	1.25	+0.16	−0.30	0.723
AILA2	3.40	1.28	−0.03	−0.71	0.714
AILA3	3.41	1.26	+0.28	−0.17	0.722
AILA4	3.41	1.23	−0.06	−0.53	0.712
**Creative engagement (**α = 0.877, ω = 0.879**, CR** = 0.879**, AVE** = 0.548**)**					
CE1	4.74	1.25	−0.11	−0.35	0.780
CE2	4.72	1.24	−0.08	−0.48	0.729
CE3	4.73	1.23	−0.23	−0.08	0.769
CE4	4.73	1.25	−0.08	−0.57	0.699
CE5	4.75	1.24	−0.18	−0.34	0.711
CE6	4.74	1.29	−0.25	−0.30	0.749
**Self-reported creative cognition (**α = 0.826, ω = 0.829**, CR** = 0.830**, AVE** = 0.496**)**					
CC1	4.57	1.27	−0.23	−0.01	0.750
CC2	4.58	1.29	−0.14	−0.47	0.741
CC3	4.59	1.25	−0.15	−0.20	0.670
CC4	4.57	1.23	−0.12	−0.20	0.747
CC5	4.57	1.26	−0.11	−0.18	0.602

Items rated on a seven-point Likert scale. λ, standardized factor loading; α, Cronbach's alpha; CR, composite reliability; AVE, average variance extracted. All loadings significant at *p* < 0.001.

**Table 2:** Descriptive statistics, correlations, and discriminant validity (*N* = 389).

Construct	*M*	*SD*	1	2	3	4	5
1. Pedagogical GenAI use	4.85	0.97	*0.715*	0.486	0.194	0.298	0.467
2. Creative self-efficacy	4.62	1.05	0.413	*0.776*	0.050	0.495	0.583
3. AI-related learning anxiety	3.41	1.00	−0.160	0.041	*0.718*	0.151	0.175
4. Creative engagement	4.73	0.98	0.256	0.429	−0.127	*0.740*	0.691
5. Self-reported creative cognition	4.57	0.97	0.389	0.492	−0.143	0.589	*0.704*

Pearson correlations below the diagonal; absolute heterotrait-monotrait (HTMT) ratios above the diagonal; square roots of AVE on the diagonal (italics). Correlations ≥|0.13| are significant at *p* < 0.01 and ≥|0.10| at *p* < 0.05; the self-efficacy-anxiety correlation is not significant. All HTMT ratios fall below the 0.85 threshold.

### 3.2 Descriptive statistics

Table 2 reports means, standard deviations, and correlations. GenAI use correlated positively with creative self-efficacy (*r* = 0.41), creative engagement (*r* = 0.26), and self-reported creative cognition (*r* = 0.39), and negatively with learning anxiety (*r* = −0.16, all *p* < 0.01). Creative engagement showed the strongest association with self-reported creative cognition (*r* = 0.59). Learning anxiety correlated near zero with self-efficacy (*r* = 0.04, n.s.), consistent with treating competence beliefs and learning worry as distinct parallel appraisals rather than opposite ends of one dimension.

### 3.3 Structural model and mediation

The structural model fit the data well, χ^2^(243) = 307.10, CFI = 0.983, RMSEA = 0.026, and explained 54% of the variance in self-reported creative cognition, 27% in creative engagement, 24% in creative self-efficacy, and 4% in learning anxiety. Figure 1 and Tables 3, 4 report the standardized estimates.

**Table 3:** Standardized structural path coefficients (std.all solution; *N* = 389).

Path	β	*SE*	*p*	95% CI
GenAI use → creative self-efficacy	0.489	0.061	< 0.001	[0.370, 0.608]
GenAI use → learning anxiety	−0.191	0.057	0.002	[−0.303, −0.079]
Creative self-efficacy → creative engagement	0.504	0.058	< 0.001	[0.390, 0.618]
Learning anxiety → creative engagement	−0.161	0.059	0.003	[−0.277, −0.045]
Creative engagement → self-reported creative cognition	0.495	0.061	< 0.001	[0.376, 0.614]
Creative self-efficacy → self-reported creative cognition	0.243	0.061	< 0.001	[0.124, 0.362]
Learning anxiety → self-reported creative cognition (n.s.)	−0.075	0.051	0.120	[−0.175, 0.025]
GenAI use → self-reported creative cognition (direct)	0.186	0.054	< 0.001	[0.080, 0.292]

Estimates are standardized (lavaan std.all). *SE*, robust standard error; *p*, exact *p*-value. Unstandardized estimates, robust standard errors, and residual covariances are reported in Supplementary Table S6, and a partial-least-squares estimator comparison in Supplementary Table S10. n.s., not significant.

**Table 4:** Standardized indirect and total associations with self-reported creative cognition (std.all; *N* = 389).

Association	β	95% CI
**Panel A. GenAI-origin indirect associations**		
GenAI → self-efficacy → cognition (H2)	0.119	[0.057, 0.189]
GenAI → anxiety → cognition (H3, n.s.)	0.014	[−0.005, 0.039]
GenAI → self-efficacy → engagement → cognition (H5a)	0.122	[0.077, 0.178]
GenAI → anxiety → engagement → cognition (H5b)	0.015	[0.003, 0.033]
Total GenAI-origin indirect association	0.270	[0.179, 0.375]
Total GenAI-use association	0.456	[0.314, 0.604]
**Panel B. Mediator-origin indirect associations (excluded from the GenAI decomposition)**		
Self-efficacy → engagement → cognition (H4)	0.249	[0.172, 0.337]
Anxiety → engagement → cognition	−0.080	[−0.144, −0.022]

Estimates are standardized (std.all). Confidence intervals are Monte Carlo intervals (200,000 draws) computed from the full covariance matrix of the path coefficients; an interval excluding zero indicates a significant association. Cognition = self-reported creative cognition. Panel A components originate at GenAI use and sum to the total GenAI-origin indirect association; Panel B associations originate at the Wave-2 mediators (H4 evaluates β = 0.249) and are not part of the GenAI decomposition. n.s., not significant.

GenAI use was associated with higher creative self-efficacy (β = 0.489, *p* < 0.001) and lower learning anxiety (β = −0.191, *p* = 0.002). Self-efficacy was associated with creative engagement (β = 0.504, *p* < 0.001), and anxiety with it negatively (β = −0.161, *p* = 0.003). Creative engagement was the strongest correlate of self-reported creative cognition (β = 0.495, *p* < 0.001), with an additional direct association from self-efficacy (β = 0.243, *p* < 0.001). The direct path from anxiety to cognition was not significant (β = −0.075, *p* = 0.12), which places the association of anxiety with cognition in the engagement channel rather than in a direct link. GenAI use retained a direct association with self-reported creative cognition after the mediators were included (β = 0.186, *p* < 0.001). Engagement was thus the largest concurrent standardized coefficient in the model, and the indirect association from self-efficacy through engagement to cognition exceeded the direct coefficient of self-efficacy, consistent with competence beliefs being associated with creative cognition mainly through sustained involvement rather than only directly.

The total modeled association between GenAI use and self-reported creative cognition was 0.456 [95% CI [0.314, 0.604]], supporting H1. The total GenAI-origin indirect association was 0.270 [0.179, 0.375], approximately 59% of the total modeled association. The specific indirect association through self-efficacy alone was 0.119 [0.057, 0.189], supporting H2. The serial association through self-efficacy and then engagement was 0.122 [0.077, 0.178], supporting the competence branch (H5a). Separately, the association originating at self-efficacy and passing through engagement to cognition was 0.249 [0.172, 0.337], supporting H4; this quantity does not include the GenAI-to-self-efficacy path and is not part of the GenAI decomposition. The anxiety routes were weaker. The simple association from GenAI use through anxiety to cognition was not significant, 0.014 [−0.005, 0.039], so H3 was not supported in its simple form. Only the serial association through anxiety and engagement was non-zero, 0.015 [0.003, 0.033], supporting the anxiety branch (H5b), and the indirect association of anxiety with cognition via engagement was likewise significant, −0.080 [−0.144, −0.022]. The anxiety branch (H5b) is thus supported, but only the engagement-involving indirect association was non-zero. Within the GenAI total-association decomposition, the direct path accounted for about 41% and the two self-efficacy components for a further 53%, while the two anxiety components together contributed about 6%. Figure 2 plots these standardized associations with their Monte Carlo intervals, distinguishing GenAI-origin components from the mediator-origin associations, and Figure 3 shows how the direct, competence, and affective components partition the total GenAI-use association.

**Figure 2:**
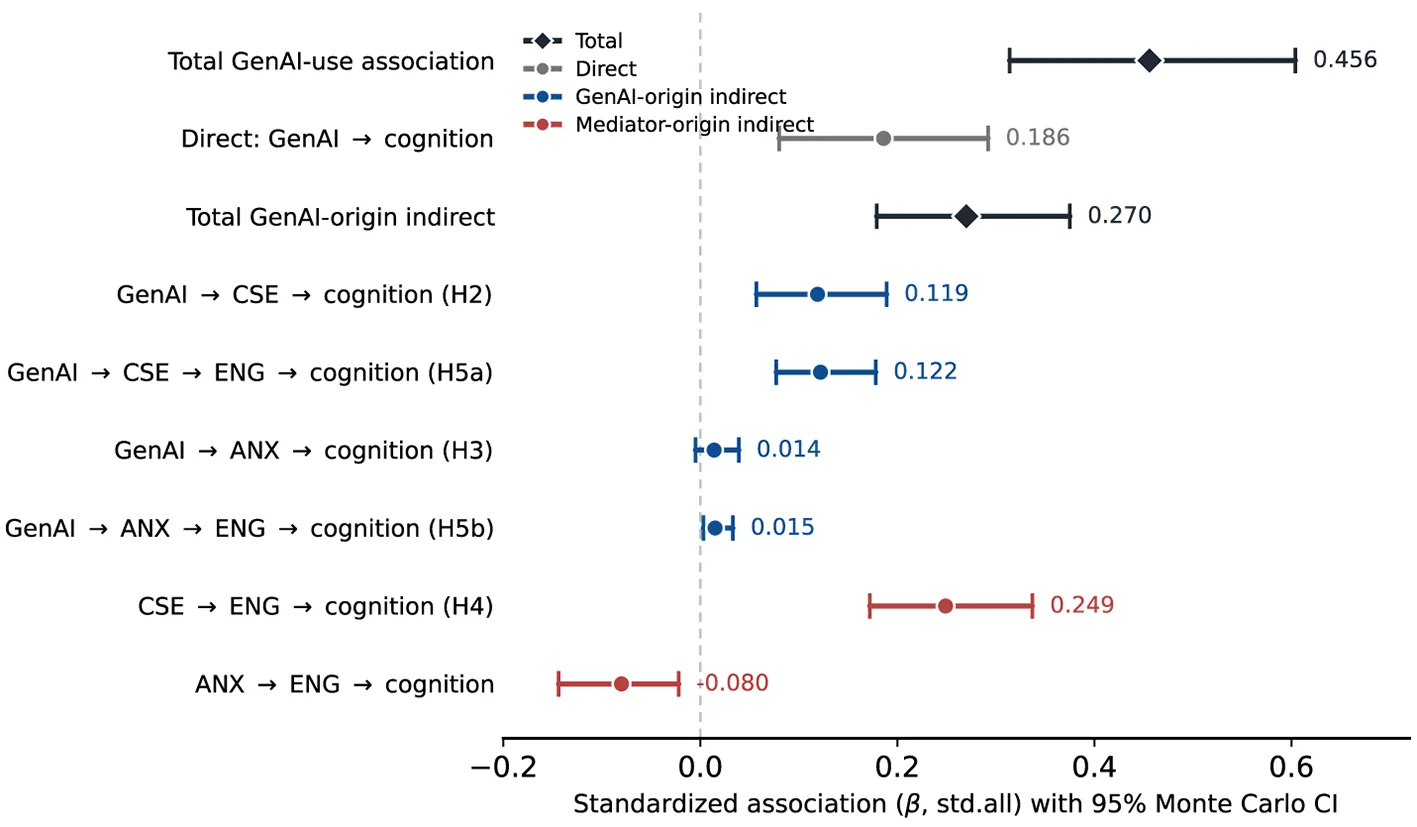
Standardized direct, indirect, and total associations with self-reported creative cognition and their 95% Monte Carlo confidence intervals (*N* = 389). Diamonds mark the total and total GenAI-origin indirect associations; circles mark component associations. GenAI-origin indirect components (blue) originate at pedagogical GenAI use, whereas mediator-origin associations (red) originate at the Wave-2 mediators and are not part of the GenAI-use decomposition. Intervals that exclude zero indicate a significant association; the simple anxiety-mediated association (H3) is the only non-significant component.

**Figure 3:**
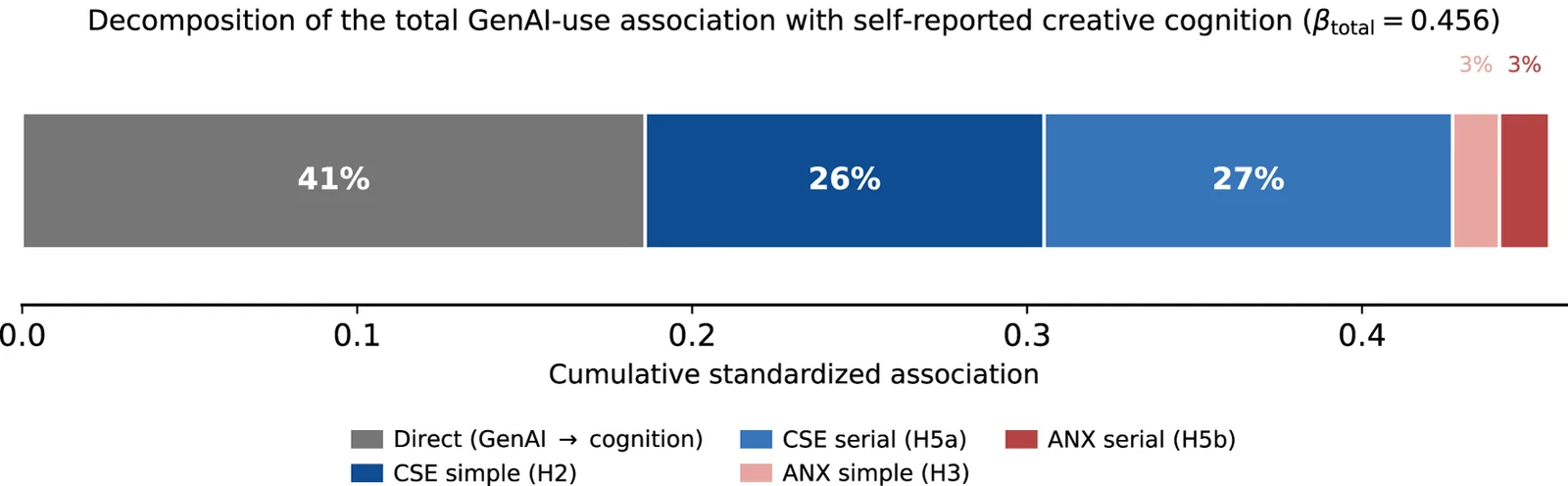
Decomposition of the standardized total GenAI-use association with self-reported creative cognition (β_total_ = 0.456) into the direct association and four GenAI-origin indirect components (*N* = 389). Percentages are shares of the total. The direct association contributes about 41%, the two self-efficacy components about 53%, and the two anxiety components about 6%.

### 3.4 Robustness

Alternative specifications did not distinguish the direction of the concurrent Wave-3 association. Adding a direct GenAI-to-engagement path left that path non-significant [β = 0.036, 95% CI [−0.066, 0.138]] and did not improve fit (ΔAIC = +1.3), and reverse (cognition-to-engagement) and non-directional models fit essentially as well as the focal model (ΔCFI ≤ 0.001; Supplementary Table S7), so the ordering of the final link is not empirically identified. The coefficient pattern was stable under the fully adjusted covariate model, WLSMV re-estimation, 10,000-draw bootstrap intervals, and exclusion of the lowest-loading cognition item (Supplementary Tables S4, S9, S10). A partial-least-squares path model on composite scores reproduced the sign and significance pattern (Supplementary Table S10), but because it shares the same directional specification and self-report source, this agreement addresses neither temporal ordering nor omitted confounding. These analyses support the stability of the coefficient pattern without identifying causal direction.

## 4 Discussion

Higher pedagogical GenAI use was associated with stronger later creative self-efficacy, lower later learning anxiety, and higher self-reported creative cognition in the specified model. About three-fifths (59%) of the total modeled association ran through the psychological states measured, with the self-efficacy-engagement branch larger than the anxiety branch. The modeled association was moderate rather than large, consistent with GenAI being one input among many into how design students report creative cognition. This pattern extends prior cross-sectional work by placing efficacy and anxiety at Wave 2 and testing engagement as the enactment channel, but should be read as evidence consistent with, rather than proof of, a causal mechanism.

The competence and affective branches were not equivalent. Creative self-efficacy showed both a direct association with self-reported cognition and an indirect association through engagement, whereas learning anxiety had no direct association and was linked to cognition only through engagement. The simple anxiety-mediated association was not significant, which means the popular framing, that AI helps creativity mainly by calming students, is not supported in this sample. Where prior cross-sectional work reported reduced anxiety as a parallel mediator of comparable standing to self-efficacy (Hwang and Wu, 2025), the Wave-2 estimates here place anxiety in a smaller, engagement-dependent role. The near-zero correlation between self-efficacy and learning anxiety reinforces that capability belief and learning worry are distinct appraisals rather than opposite ends of one continuum. However, engagement and cognition were measured concurrently, and reverse and non-directional specifications fit essentially as well as the focal model, so the data establish a robust association between these Wave-3 constructs but not its direction.

For teaching, the results support testable propositions rather than prescriptions. Tasks that require students to compare alternatives, document iterations, and justify revisions may be more likely to combine GenAI use with active engagement than tasks that reward rapid output alone, and process-based assessment that credits iteration and rationale may support creative cognition more than grading a final artifact; because the study did not manipulate task framing, assessment, or feedback, both are hypotheses for intervention studies rather than established effects. Future work should manipulate task framing and compare productive, passive, and over-reliant forms of use, and should distinguish text, image-generation, and editing tools, required from optional use, and ideation support from generation of final artifacts. Because our outcome is self-reported individual cognition, it also remains possible that individual gains coincide with convergence across a cohort, so direct measurement of solution diversity at the cohort level is a useful complement (Doshi and Hauser, 2024; Anderson et al., 2024).

Several limitations qualify the conclusions. All measures were self-reports from one institution in one country, so the estimates may not transfer to other educational systems or to programs with different tool cultures, and self-reported creative cognition was not corroborated by rubric-rated or expert-rated work. The study was observational and did not adjust for baseline levels of the mediators or the outcome; unmeasured factors such as prior creative achievement, digital competence, instructional quality, or self-selection into GenAI use could shape both tool use and the mediators, and frequent use may itself mark more supportive instruction rather than act as its source. A linear average association also cannot exclude threshold or inverted-U effects of heavier or over-reliant use. Because all constructs came from the same respondents and the outcome was self-reported, and because engagement and cognition were concurrent, the common-latent-factor analysis is a sensitivity check rather than proof that common-method variance is absent. The aggregation of heterogeneous functions into one use score and the marginal AVE of the cognition scale further limit interpretation, and the results may not extend to performance-based art forms such as music or dance. Replications should use logged tool behavior, rubric- or expert-rated creative products, baseline-adjusted or experimental designs, and separate measurement occasions for engagement and cognition. Within these limits, the findings are consistent with an appraisal-to-engagement account in which competence beliefs and creative engagement constituted most of the modeled indirect association, while reduced learning anxiety plays only a small, engagement-dependent part.

## Data Availability

The raw data supporting the conclusions of this article will be made available by the authors, without undue reservation.
