# Supplementary material for: Pedagogical GenAI use and self-reported creative cognition in art education: a three-wave time-lagged correlational study among design students
Source: Front Psychol. 2026 Oct 6;17:1916135. doi: 10.3389/fpsyg.2026.1916135 (PMC13680391; doi:10.3389/fpsyg.2026.1916135)
Supplement: Supplementary file 1 [file Data_Sheet_1.pdf]

Supplementary Material

1 OVERVIEW

This Supplementary Material provides the reproducibility, measurement, and robustness detail summarized in the main report. It contains the complete measurement model (Figure S1), the participant-flow diagram (Figure S2), the full set of administered items with their Korean and English wording and their source-to-item adaptation (Tables S2–S3), and the estimation, alternative-model, power, covariate, and sensitivity analyses referenced in the Methods and Results (Tables S1 and S4–S10). Because the analytic sample contained no item-level missing data, all models were estimated by complete-case robust maximum likelihood (MLR) in lavaan, with seven-point items treated as continuous, each latent variable scaled by fixing its first loading, and the Wave-2 disturbances of creative self-efficacy and learning anxiety allowed to covary. Standardized coefficients use the `std.all` solution, and Monte Carlo intervals (200,000 draws) are computed from the full estimated covariance matrix of the path coefficients.

2 SUPPLEMENTARY FIGURES

3 SUPPLEMENTARY TABLES

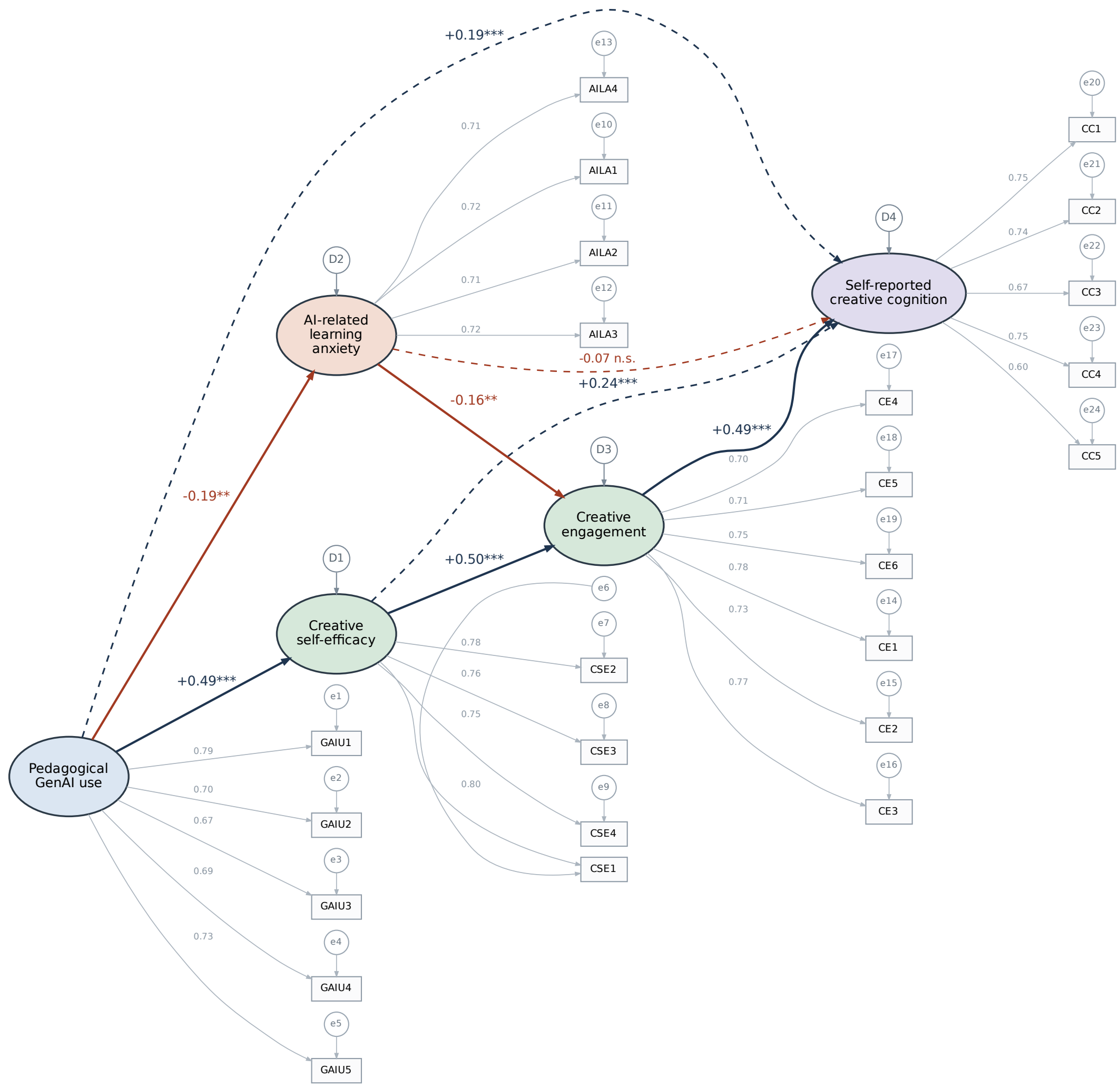

Figure S1. Complete measurement and structural model with standardized estimates ($N = 389$). Latent constructs are drawn as ellipses and their measured indicators as rectangles; e_1–e_{24} are indicator measurement errors and D_1–D_4 are the structural disturbances of the endogenous constructs. Values on the loading arrows are standardized factor loadings. Solid arrows are the hypothesized structural paths and dashed arrows are the direct paths estimated with the mediators included. This complete diagram replaces the dense version formerly shown in the main text.

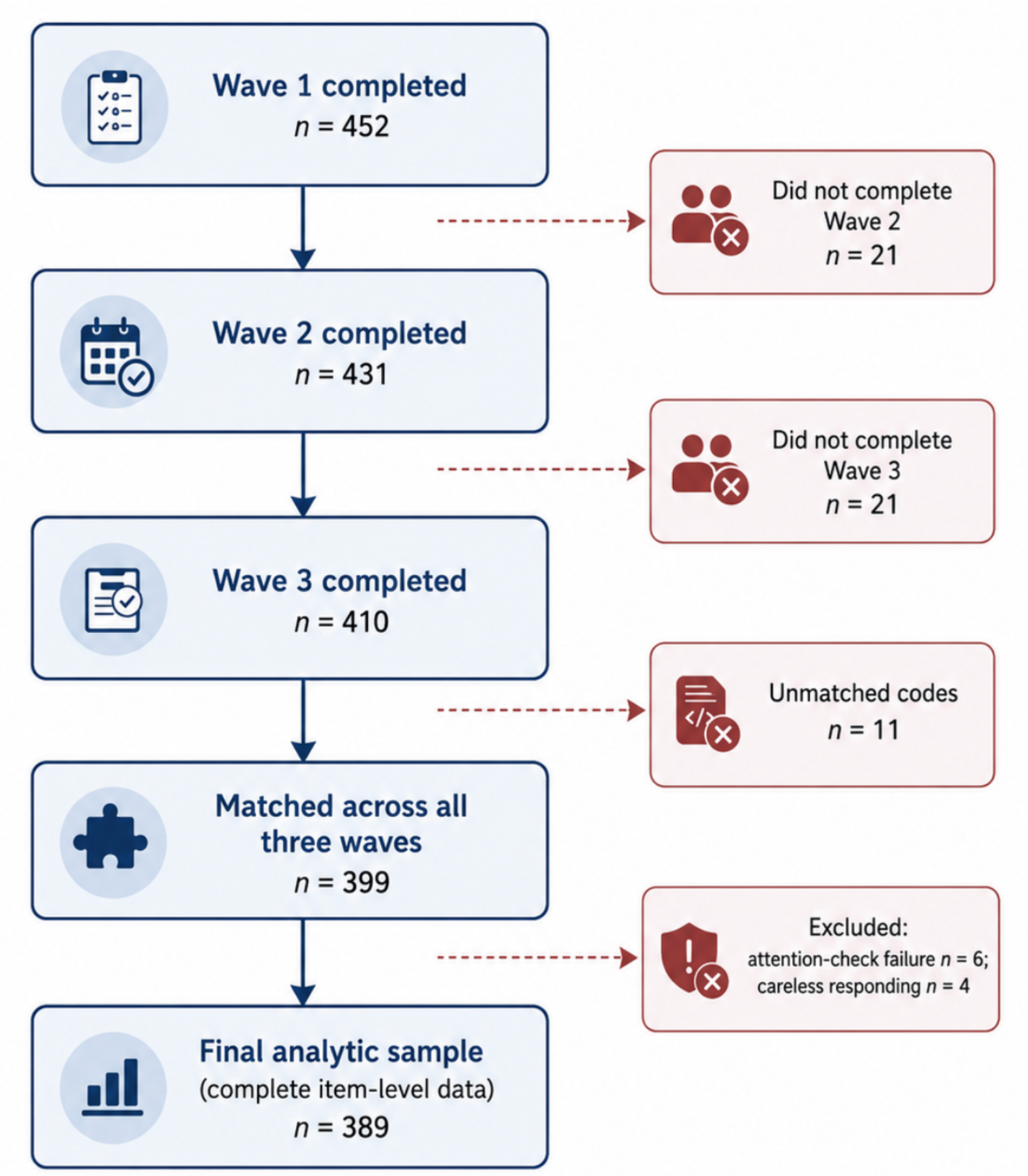

Figure S2. Participant flow across the three waves, matching, and screening. Of 452 Wave-1 respondents, 431 completed Wave 2 and 410 completed Wave 3; 399 records matched across all three waves through self-generated anonymous codes. From the matched records, 6 cases failing an embedded attention-check item and 4 cases flagged for careless responding were removed, leaving 389 cases with complete item-level data.

Table S1. Recruitment context, participant flow, and logistic attrition analysis.

Panel A. Recruitment and screening context	
Setting	Undergraduate design majors, one public university, Busan, Republic of Korea
Recruitment	Central departmental announcement (not by course instructors) during one semester
Waves	Three waves spaced ≈2 weeks apart within the same semester
Incentive	Voluntary participation; not linked to course grades
Linkage	Self-generated anonymous codes; no names, student numbers, or contacts collected
Attention check	One embedded instructed-response item ("Please select *strongly agree* for this item"); failure = incorrect response
Careless responding	Combined screen: longest-string $\geq$ 10 identical consecutive responses *or* completion time < 2 s per item
Missing data	Analytic sample retained only complete item-level cases; no imputation required
Tool/platform use	Survey recorded coursework *functions* of use; platform-specific tools were not recorded (see Limitations)
Use requirement and instructional context	Required versus optional use, instructor guidance, institutional AI-use rules, and use in final artefacts were not recorded
Class/instructor identifiers	Not available; class-level clustering could not be evaluated and is noted as a limitation

Panel B. Participant flow	
Wave 1 completed	$n = 452$
Wave 2 completed	$n = 431$
Wave 3 completed	$n = 410$
Matched across all three waves	$n = 399$
Excluded: attention-check failure	$n = 6$
Excluded: careless responding	$n = 4$
Final analytic sample	$n = 389$ (86.1% of Wave 1)

Panel C. Logistic attrition model (outcome: retained in final sample = 1)

Wave-1 predictor	OR	95% CI	p
Pedagogical GenAI use	1.08	[0.82, 1.42]	0.59
Female	0.94	[0.54, 1.64]	0.83
Year of study	1.05	[0.85, 1.30]	0.65
Prior duration of GenAI use	1.11	[0.87, 1.42]	0.40
Age	0.98	[0.86, 1.11]	0.74

Nagelkerke $R^2 = 0.01$; model $\chi^2(5) = 2.6$, $p = 0.76$. All standardized mean differences between retained and attrited respondents on Wave-1 variables were < 0.15, indicating no material selective attrition on the study variables.

Table S2. Complete administered items (English and Korean), construct, item code, and source-to-item adaptation (Part 1 of 2: pedagogical GenAI use, creative self-efficacy, and AI-related learning anxiety).

**Code**	**English wording**	Korean wording (administered)
Pedagogical GenAI use — study-specific measure developed for this coursework context		
GAIU1	I use generative AI tools to generate initial ideas for coursework design tasks.	나는 수업 디자인 과제의 초기 아이디어를 얻기 위해 생성형 AI 도구를 사용한다.
GAIU2	I use generative AI tools to explore alternative visual styles for my design assignments.	나는 디자인 과제를 위해 대안적인 시각적 스타일을 탐색하려고 생성형 AI 도구를 사용한다.
GAIU3	I use generative AI tools to revise or refine my design work through prompts.	나는 프롬프트를 통해 디자인 작업을 수정하고 다듬기 위해 생성형 AI 도구를 사용한다.
GAIU4	I use generative AI tools to compare multiple design alternatives before deciding.	나는 결정을 내리기 전에 여러 디자인 대안을 비교하려고 생성형 AI 도구를 사용한다.
GAIU5	I use generative AI tools to obtain feedback on drafts of my coursework.	나는 과제 초안에 대한 피드백을 얻기 위해 생성형 AI 도구를 사용한다.
Creative self-efficacy — adapted from Tierney and Farmer (2002)		
CSE1	I feel confident in my ability to generate novel ideas in my design coursework.	나는 디자인 수업에서 새로운 아이디어를 만들어 낼 능력이 있다고 확신한다.
CSE2	I have confidence in my ability to develop others' ideas further in design tasks.	나는 디자인 과제에서 다른 사람의 아이디어를 더 발전시킬 자신이 있다.
CSE3	I am good at finding creative solutions to design problems in my coursework.	나는 수업의 디자인 문제에 대해 창의적인 해결책을 잘 찾는다.
CSE4	I trust my talent and skills for creative work in design.	나는 디자인 창작 작업에 대한 나의 재능과 능력을 믿는다.
AI-related learning anxiety — adapted from learning-anxiety subscale of Wang and Wang (2022)		
AILA1	I worry that relying on AI tools will weaken my own creative skills in coursework.	나는 AI 도구에 의존하면 나의 창의적 역량이 약해질까 봐 걱정된다.
AILA2	I feel anxious about whether AI-assisted design work is truly my own.	나는 AI의 도움을 받은 디자인 작업이 진정으로 나의 것인지 불안하다.
AILA3	I am concerned about keeping pace with classmates in using AI tools for design.	나는 디자인에 AI 도구를 활용하는 데 있어 동료들을 따라가지 못할까 봐 염려된다.
AILA4	Learning to use AI tools for creative coursework makes me feel uneasy.	창의적 과제를 위해 AI 도구 사용법을 배우는 것이 나를 불안하게 한다.

Table S2. (Continued.) Part 2 of 2: creative engagement and self-reported creative cognition.

**Code**	**English wording**	Korean wording (administered)
Creative engagement — assembled from Fredricks et al. (2004) and Zhang and Bartol (2010)		
CE1	I put a great deal of effort into the creative tasks in my design coursework.	나는 디자인 수업의 창의적 과제에 많은 노력을 기울인다.
CE2	I persist with design tasks even when they become difficult.	나는 디자인 과제가 어려워져도 끝까지 해낸다.
CE3	I feel enthusiastic when working on creative design tasks.	나는 창의적인 디자인 과제를 할 때 열정을 느낀다.
CE4	I become absorbed and lose track of time when I work on design tasks.	나는 디자인 과제에 몰입하여 시간 가는 줄 모른다.
CE5	I spend considerable time searching for information relevant to a design problem.	나는 디자인 문제와 관련된 정보를 찾는 데 상당한 시간을 쓴다.
CE6	I try many different perspectives before settling on a design solution.	나는 디자인 해결책을 정하기 전에 여러 관점을 시도한다.
Self-reported creative cognition — study-specific measure informed by Finke et al. (1992)		
CC1	In my design coursework, I generate many different ideas for a given brief.	나는 디자인 과제에서 주어진 주제에 대해 다양한 아이디어를 만들어 낸다.
CC2	I combine unrelated concepts to create new design ideas.	나는 서로 관련 없는 개념들을 결합하여 새로운 디자인 아이디어를 만든다.
CC3	I explore unconventional possibilities when developing a design.	나는 디자인을 발전시킬 때 관습에 얽매이지 않는 가능성을 탐색한다.
CC4	I reinterpret design problems in original ways.	나는 디자인 문제를 독창적인 방식으로 재해석한다.
CC5	I mentally transform and reorganize visual elements when developing ideas.	나는 아이디어를 발전시킬 때 시각적 요소들을 머릿속에서 변형하고 재구성한다.

All items used a seven-point Likert response (1 = strongly disagree, 7 = strongly agree). “Adapted” indicates items reworded from a published scale for the design-coursework context; “study-specific / informed by” indicates items written for this study and guided by, rather than taken from, the cited framework. CC5 is the lowest-loading cognition item ($\lambda = 0.602$) and is examined in the deletion sensitivity of Table S4. The Korean wording is the administered version; the English wording is the reconciled forward/back translation (Table S3).

Table S3. Translation, expert review, pilot, and post hoc content-classification audit.

Step	Procedure and outcome
Original language	Items were finalized in Korean, the language of instruction and administration.
Translation direction	English drafts (developed and adapted) were translated into Korean and independently back-translated into English.
Translators	Two bilingual translators with graduate training in design/education produced the forward and back translations independently; a third bilingual reviewer, not involved in item writing, adjudicated discrepancies.
Reconciliation	Discrepancies were resolved by consensus; two items were reworded for idiomatic clarity before piloting.
Expert review	Two design educators, independent of the research team, rated each item for relevance to studio coursework on a 4-point scale and classified it by intended construct.
Content validity	Item-level content validity index (I-CVI) ≥ 0.80 for 22 of 24 items; scale-level S-CVI/Ave $= 0.93$; primary-construct classification agreement $= 87\%$.
Pilot sample	38 design students (not in the main sample; 63% women; $M_{\text{age}} = 21.6$) completed the instrument and rated clarity.
Pilot statistics	Cronbach's α ranged 0.79–0.88 across the five scales; no item reduced its scale's α if deleted; median clarity rating $= 6$ of 7.
Post hoc content audit	Five independent design-education and psychometric experts sorted items into capability belief, engagement, cognitive operation, or outcome evaluation. Agreement with intended category $= 84\%$; CC5 was classified by 2 of 5 experts as a capability/outcome rather than a cognitive-operation item, motivating the deletion sensitivity in Table S4.

The content audit was conducted at the revision stage and is a content check, not a pre-administration validation. Discriminant statistics (HTMT ≤ 0.69) indicate statistical separation but do not by themselves resolve conceptual overlap or shared self-perception.

Table S4. Additional measurement diagnostics, CC5-deletion sensitivity, and WLSMV re-estimation.

Diagnostic	GAIU	CSE	AILA	CE	CC
Cronbach's α	0.839	0.858	0.810	0.877	0.826
McDonald's ω	0.840	0.859	0.811	0.879	0.829
Composite reliability	0.839	0.858	0.810	0.879	0.830
AVE	0.511	0.602	0.516	0.548	0.496

Additional diagnostics and sensitivities

Analysis	Result
Largest absolute standardized residual	0.082 (below 0.10)
Largest modification index (cross-loading)	8.7 (CE4 on cognition); not added, no substantive cross-loading
CC5 deleted (four-item cognition scale)	AVE $= 0.530$, CR $= 0.818$; focal structural coefficients changed by ≤ 0.02
WLSMV re-estimation (ordered items)	CFI $= 0.982$, TLI $= 0.980$, RMSEA $= 0.031$, SRMR $= 0.047$
Competence serial (β, 95% CI)	0.118 [0.072, 0.174]
Anxiety serial (β, 95% CI)	0.014 [0.002, 0.031]

The convergent validity of self-reported creative cognition is marginal (AVE $= 0.496$; lowest loading $= 0.602$). Deleting CC5 raises AVE above 0.50 but lowers reliability; the five-item scale is therefore retained as primary and the four-item version reported only as a sensitivity.

Table S5. Common-method assessment: Harman's diagnostic and common-latent-factor (CLF) model.

Element	Specification / result
Harman's single-factor test (ancillary only)	One unrotated factor accounted for 30.5% of the variance ($< 50\%$); reported only as a weak diagnostic
CLF specification	Every indicator loaded on both its substantive trait and one orthogonal (uncorrelated) method factor
Identification	Method-factor loadings constrained equal across indicators to identify the common variance component
Convergence and fit	CLF model converged; CFI $= 0.984$, RMSEA $= 0.025$
Average method variance	$\approx 8\%$ of indicator variance attributable to the method factor
Impact on structural paths	Largest change in a focal standardized coefficient $= 0.03$; all signs and significance retained
Interpretation	Common-source variance is reduced but not eliminated; because the outcome is self-reported and engagement and cognition are concurrent, common-method bias remains a limitation

Table S6. Full structural estimates: unstandardized (B), robust SE, standardized (β, `std.all`), exact p, and 95% CI.

Path	B	SE_B	β	p	95% CI (β)
GenAI use → creative self-efficacy	0.529	0.066	0.489	< 0.001	[0.370, 0.608]
GenAI use → learning anxiety	−0.197	0.059	−0.191	0.002	[−0.303, −0.079]
Creative self-efficacy → creative engagement	0.470	0.054	0.504	< 0.001	[0.390, 0.618]
Learning anxiety → creative engagement	−0.158	0.058	−0.161	0.003	[−0.277, −0.045]
Creative engagement → creative cognition	0.490	0.060	0.495	< 0.001	[0.376, 0.614]
Creative self-efficacy → creative cognition	0.225	0.056	0.243	< 0.001	[0.124, 0.362]
Learning anxiety → creative cognition	−0.073	0.049	−0.075	0.120	[−0.175, 0.025]
GenAI use → creative cognition (direct)	0.186	0.054	0.186	< 0.001	[0.080, 0.292]
CSE ↔ anxiety (Wave-2 disturbance cov., std.)	—	—	0.120	0.041	[0.005, 0.233]

Robust (MLR) estimation; scaling correction factor $= 1.08$; robust $\chi^2(243) = 307.10$, CFI $= 0.983$, TLI $= 0.981$, RMSEA $= 0.026$, SRMR $= 0.044$. The model includes the Wave-2 disturbance covariance of creative self-efficacy and learning anxiety (final row). Unstandardized estimates are in the observed seven-point metric.

Table S7. Alternative structural specifications and fit comparison ($N = 389$).

Model	robust χ^2(df)	CFI	TLI	RMSEA	SRMR	ΔAIC
M0: focal model (incl. CSE ↔ anxiety residual covariance)	307.10 (243)	0.983	0.981	0.026	0.044	0.0
M1: M0 + GenAI → engagement	306.35 (242)	0.983	0.982	0.026	0.043	$+1.3$
M2: reverse Wave-3 ordering (cognition → engagement)	307.30 (243)	0.983	0.981	0.026	0.044	$+0.2$
M3: nondirectional Wave-3 association (engagement ↔ cognition residual)	307.00 (243)	0.983	0.982	0.026	0.044	-0.1
M4: reciprocal engagement ↔ cognition	not identified without exclusion restrictions					—

M0 is the focal model reported in the main text; it already includes the Wave-2 disturbance covariance of creative self-efficacy and learning anxiety (standardized $= 0.12$, $p = 0.04$; Table S6). The added direct path in M1 was non-significant ($\beta = 0.036$, 95% CI [−0.066, 0.138], $p = 0.49$) and did not improve fit. M2 and M3 fit essentially as well as M0 (ΔCFI ≤ 0.001), so the direction of the concurrent Wave-3 engagement–cognition link is not empirically identified. M4 is reported as unidentified rather than interpreted.

Table S8. Model-based Monte Carlo power and interval coverage for the principal indirect associations (10,000 replications).

Target indirect association	β	Power	95% CI coverage
GenAI → CSE → cognition	0.119	0.94	0.95
GenAI → anxiety → cognition	0.014	0.24	0.94
GenAI → CSE → engagement → cognition	0.122	0.99	0.95
GenAI → anxiety → engagement → cognition	0.015	0.52	0.94
CSE → engagement → cognition	0.249	> 0.99	0.95
Anxiety → engagement → cognition	−0.080	0.73	0.95

Data were generated from the fitted model (paths, loadings, residuals, $N = 389$, MLR, no item-level missingness). Power is the proportion of replications whose Monte Carlo interval excluded zero. The competence serial association is well powered, whereas the small anxiety associations are underpowered, so their point estimates should be read cautiously.

Table S9. Fully adjusted covariate model (theoretically selected covariates).

Path	Unadjusted β	Adjusted β
GenAI use → creative self-efficacy	0.489	0.476
GenAI use → learning anxiety	−0.191	−0.184
Creative self-efficacy → creative engagement	0.504	0.497
Learning anxiety → creative engagement	−0.161	−0.154
Creative engagement → creative cognition	0.495	0.486
Creative self-efficacy → creative cognition	0.243	0.237
Learning anxiety → creative cognition	−0.075	−0.069
GenAI use → creative cognition (direct)	0.186	0.178
GenAI → CSE → engagement → cognition	0.122	0.115
GenAI → anxiety → engagement → cognition	0.015	0.014

Covariates (age, gender, year of study, major, and prior duration of GenAI use) were allowed to correlate with GenAI use and to predict all endogenous constructs. They were included on theoretical grounds rather than selected by their effect on the coefficients. Signs and significance were unchanged. Unmeasured confounders (prior creative achievement, academic performance, general digital competence, instructional quality, course/instructor effects) remain possible and are acknowledged in the Discussion.

Table S10. Bootstrap intervals, PLS estimator comparison, and unmeasured-confounding sensitivity.

Panel A. Nonparametric bootstrap (10,000 resamples)

Association	β	Bootstrap 95% CI
GenAI → CSE → cognition	0.119	[0.058, 0.190]
GenAI → anxiety → cognition	0.014	[−0.006, 0.039]
GenAI → CSE → engagement → cognition	0.122	[0.077, 0.180]
GenAI → anxiety → engagement → cognition	0.015	[0.003, 0.034]
CSE → engagement → cognition	0.249	[0.174, 0.341]

Panel B. PLS-SEM estimator comparison (composite scores)

Path	CB-SEM β	PLS β
GenAI use → creative self-efficacy	0.489	0.413
GenAI use → learning anxiety	−0.191	−0.160
Creative self-efficacy → creative engagement	0.504	0.435
Learning anxiety → creative engagement	−0.161	−0.145
Creative engagement → creative cognition	0.495	0.434
Creative self-efficacy → creative cognition	0.243	0.239
Learning anxiety → creative cognition	−0.075	−0.071
GenAI use → creative cognition (direct)	0.186	0.168

Panel C. Sensitivity to an unmeasured confounder of the mediation paths

Path	Approx. $\lvert\rho\rvert$ nullifying the association
GenAI → CSE → engagement → cognition (competence serial)	$\approx$ 0.30–0.35
GenAI → anxiety → engagement → cognition (anxiety serial)	$\approx$ 0.08–0.10

PLS coefficients are attenuated relative to the latent-variable solution, as expected when measurement error is not modeled; because PLS shares the same directional specification and self-report source, agreement is not evidence against alternative orderings or omitted confounding. Panel C uses composite scores and is a diagnostic only: the competence serial association is robust to moderate unmeasured confounding, whereas the small anxiety serial association is sensitive to weak confounding.
